# Retraction Note: Nrf2 overexpression increases the resistance of acute myeloid leukemia to cytarabine by inhibiting replication factor C4

**DOI:** 10.1038/s41417-025-00913-9

**Published:** 2025-05-08

**Authors:** Tianzhen Hu, Chengyun Pan, Tianzhuo Zhang, Ming Ni, Weili Wang, Siyu Zhang, Ying Chen, Jishi Wang, Qin Fang

**Affiliations:** 1 College of Pharmacy, Guizhou Medical University, Guiyang, Guizhou China; 2 Department of Haematology, Affiliated Hospital of Guizhou Medical University, Guizhou Province Institute of Hematology, Guiyang, Guizhou China; 3 School of Basic Medical Sciences, Guizhou Medical University, Guiyang, Guizhou China; 4 pharmacy department, Affiliated Hospital of Guizhou Medical University, Guiyang, Guizhou China

Retraction to: *Cancer Gene Therapy* 10.1038/s41417-022-00501-1, published online 15 July 2022

The Editor-in-Chief has retracted this article. After publication, concerns were raised regarding the animal study, discrepancies between figures and their corresponding supplementary data, and highly similar images within the article. Specifically:

1. Fig. 3E, THP-1, L-Nrf2 and Fig. 3E L-Nrf2 Ara-C(uM)
2. Fig. 4B, Nrf2 (THP-1) and Suppl Western blot Figure 3A, B, repeat 2 Nrf2 (EV2) (MV4-11)
3. Suppl Western Blot Figure 3A, B, repeat 2, Nrf2, EV2 (MV4-11) and Suppl Western Blot Figure 4A, B Repeat 2, Nrf2 EV2 (THP-1)
4. Suppl Western Blot Figure 4A, B, Repeat3, RFC4 (TPH-1) and Suppl Western Blot Figure 4A, B, Repeat1, RFC4 EV1 (THP-1)
5. Suppl Western Blot Figure 4A, B, Repeat2, Nrf2 EV1(U937) and Suppl Western Blot Figure 4A, B, Repeat2 Nrf2 EV2 (U937)
6. Suppl Western Blot Figure 4C, D, Repeat3, B-actin EV1 (TPH-1) and Suppl Western Blot Figure 4C, D, Repeat2 B-actin EV2 (TPH-1)
7. Suppl Western Blot Figure 4C, D, Repeat3, RFC4 EV1 (TPH-1) and Suppl Western Blot Figure 4C, D, Repeat2 B-actin EV2 (U937) and Suppl Western Blot Figure 4C, D, Repeat1, RFC4 EV1 (MV4-11)
8. Suppl Western Blot Figure 4C, D, Repeat2, RF4 EV1 (U937) and Suppl Western Blot Figure 4C, D, Repeat3 RF4 EV1 (MV4-11)
9. Suppl Western Blot Figure 7, Repeat2, p-JNK (TPH-1) and Suppl Western Blot Figure 7, Repeat3, p-JNK (TPH-1)
10. Supplementary Figure 1C, CR1 (Merge) and Supplementary Figure 1C CR5 (Merge)

The authors admitted the errors and requested a correction. However, due to the number of errors, the Editor-in-Chief no longer has confidence in the presented data.

Q. Fang disagrees with this retraction. Y. Chen, T. Hu, M. Ni, C. Pan, J. Wang, W. Wang, T. Zhang, S. Zhang did not respond to correspondence from the Publisher about this retraction.

